# Design and Fabrication of Silicon-Blazed Gratings for Near-Infrared Scanning Grating Micromirror

**DOI:** 10.3390/mi13071000

**Published:** 2022-06-25

**Authors:** Sinong Zha, Dongling Li, Quan Wen, Ying Zhou, Haomiao Zhang

**Affiliations:** 1 Key Laboratory of Optoelectronic Technology and System of the Education Ministry of China, Chongqing University, Chongqing 400030, China; c18722576829@163.com (S.Z.); quan.wen@cqu.edu.cn (Q.W.); 201908131093@cqu.edu.cn (H.Z.); 2 National Key Laboratory of Fundamental Science of Novel Micro/Nano Device and System Technology, Chongqing University, Chongqing 400030, China; 3 Chongqing Chuanyi Automation Co., Ltd., Chongqing 401121, China; zhouying314422@163.com

**Keywords:** silicon-blazed grating, platform and fillet, anisotropic wet etching, oxidation sharpening, diffraction efficiency

## Abstract

Blazed gratings are the critical dispersion elements in spectral analysis instruments, whose performance depends on structural parameters and topography of the grating groove. In this paper, high diffraction efficiency silicon-blazed grating working at 800–2500 nm has been designed and fabricated. By diffraction theory analysis and simulation optimization based on the accurate boundary integral equation method, the blaze angle and grating constant are determined to be 8.8° and 4 μm, respectively. The diffraction efficiency is greater than 33.23% in the spectral range of 800–2500 nm and reach the maximum value of 85.62% at the blaze wavelength of 1180 nm. The effect of platform and fillet on diffraction efficiency is analyzed, and the formation rule and elimination method of the platform are studied. The blazed gratings are fabricated by anisotropic wet etching process using tilted (111) silicon substrate. The platform is minished by controlling etching time and oxidation sharpening process. The fillet radius of the fabricated grating is 50 nm, the blaze angle is 7.4°, and the surface roughness is 0.477 nm. Finally, the blazed grating is integrated in scanning micromirror to form scanning grating micromirror by MEMS fabrication technology, which can realize both optical splitting and scanning. The testing results show that the scanning grating micromirror has high diffraction efficiency in the spectral range of 810–2500 nm for the potential near-infrared spectrometer application.

## 1. Introduction

Diffraction gratings are widely used as core optical splitters in modern optical devices such as ultra-precision measurement systems [1,2], spectrometers [3,4,5,6], semiconductor lasers [7,8], display techniques [9,10], and so on. With the development of micro-electro-mechanical system (MEMS) technology, there are kinds of silicon diffraction gratings developed, including rectangle gratings, sine gratings, and blazed gratings. Particularly, the blazed gratings exhibit excellent optical characteristics since they can focus the major part of the incident energy on a single non-zero diffraction order, which is beneficial for spectral analysis and detection. Generally, the diffraction and spectral characteristics of the blazed gratings are primarily dependent on the shape of the grating grooves, such as blaze angle and the topography of the grating groove. Therefore, it is important to design a proper blaze angle in a certain spectral range and control the grating shape during fabrication. For example, Zamkotsian et al. designed blazed gratings with a blaze angle of 5.04° working at the spectral range of 400–800 nm for high throughput spectrographs in space missions [11]. Sokolov et al. proposed blazed gratings with a blaze angle of 0.6°, 0.8°, and 1.0°, respectively, to achieve high diffraction efficiency at the tender X-ray region [12].

Mechanical ruling technique [12,13,14,15,16], ion beam etching (IBE) technique [17,18,19,20,21,22], and anisotropic wet etching technique [23,24,25] are commonly used to fabricate silicon-blazed gratings. Mechanical ruling technique using a specific shape diamond cutting tool extrudes on the grating substrate coated with aluminum or gold film to form grating grooves, which is simple and low-cost. But this method is not suitable for batch manufacturing of large area grating due to its low efficiency. Moreover, it is not compatible with MEMS fabrication process, and it is difficult to fabricate gratings on movable MEMS devices. During the IBE process, the grating structure is shaped through physical sputtering of materials. The blazed gratings with different blaze angle can be fabricated by adjusting the incident angle of ion beam, the duty cycle of the photoresist mask, and the etching rate ratio of the photoresist to the substrate material, which has the advantages of good direction, speed and shape control, high precision and smooth grating surface can be achieved [17,18]. Nevertheless, the fabrication process depends on the IBE equipment, so it is complex and costly. Furthermore, the etching rate of IBE is very low, and it is also not suitable for batch preparation of gratings. Anisotropic wet etching is the most promising technique for fabrication of high-performance blazed grating. It is a simple and convenient method to realize arbitrary blazed angle by using tilted (111) silicon wafers. Moreover, it provides atomically smooth blazed facets since it is formed by (111) lattice plane. However, there is always a small platform or nub left on the top of grating groove after etching, which will lead to amounts of stray light and reduce the diffraction efficiency. Voronov et al. fabricated 6° tilted blazed facets grating grooves by KOH solution, but there were silicon nubs on its top [26]. So, they used piranha solution (H_2_SO_4_ + H_2_O_2_) to chemically oxidize the silicon nubs, and then sharpened the gratings by hydrofluoric acid (HF) removal of the resulting oxides. However, there were 26 cycles applied to remove the 25-nm wide nubs in total, leading to complex fabrication process and large surface roughness. Fr$\ddot{u}$hauf and Kr$\ddot{o}$nert prepared triangular grating grooves with 500 nm width platform by anisotropic wet etching process using tetramethylammonium hydroxide (TMAH) solution [27], then the grating grooves were etched for a short time by isotropic etching (mixed solution of HNO_3_, HF, and CH_3_COOH). Finally, the radius of the convex edge was diminished to 50 nm, but the surface of the triangular gratings was relatively rough. Miles et al. fabricated a master blazed grating with platform width of 30 nm by combining electron beam etching and anisotropic wet etching [28]. To maximize the diffraction efficiency, the master grating was replicated using UV nanoimprinting lithography with the result that the platform became a flat trough which was small enough to be shadowed by the neighboring facet. However, gratings made on the imprint resist are limited in applications compared with those made on the silicon substrate. In our previous work, an 800–1800 nm blazed grating whose blaze angle is 7.4° was prepared by anisotropic wet etching and oxidation sharpening, but the width of the platform was 540 nm and there was a lack of systematic optimization research on etching and sharpening process [29].

The purpose of this paper is to design and fabricate high performance silicon-blazed gratings for near-infrared scanning grating micromirror used in miniature spectrometers. First, the blazed grating working at wavelength range from 800 nm to 2500 nm was designed. Effect of platform and fillet on diffraction efficiency is simulated and optimized. Reactive ion etching (RIE) and wet isotropic etching are investigated to prepare SiO_2_ mask and narrow its width. The grating groove is formed by anisotropic wet etching of tilted (111) silicon substrate, and then sharpened by high temperature oxidation sharpening process. The optimal anisotropic wet etching time and oxidation sharpening time was obtained, and the grating grooves were analyzed by optical profiler, scanning electron microscope (SEM), and atomic force microscope (AFM). Finally, the blazed grating is integrated in scanning micromirror by MEMS fabrication technology to form scanning grating micromirror, and the optical properties are tested by diffraction efficiency measurement system and diffracted wavelength range test system.

## 2. Design of the Grating Structure

Performance of the blazed grating is quite dependent on its groove parameters, including blaze angle and grating constant. In this section, based on diffraction theory and diffraction efficiency simulation, the blazed grating structure is determined.

### 2.1. Principles

**Figure 1:**
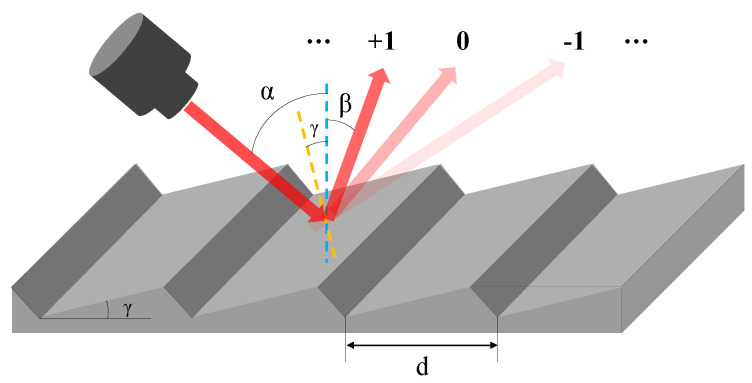
Schematic and optical path diffraction of a blazed grating.

Blazed grating exhibits excellent optical characteristics because it can concentrate light energy at a specific diffraction order through diffraction plane at a specific blaze angle. The blazed grating and the optical path of its diffraction are depicted in Figure 1.

The monochromatic light obliquely incident on the blazed grating, and then it is diffracted into discrete directions. Each grating profile can be considered to be a small, slit-shaped source of diffracted light that combines to form a set of diffracted wave fronts. Because the diffracted lights from different grating surfaces have the same phase, thus resulting in constructive interference. The relationships of incident angle *α*, diffraction angle *β*, grating constant *d* and the incident light wavelength *λ* are given by the diffraction grating equation, as shown in Equation (1):

(1) $$d\left(\sin\alpha -\sin\beta \right)=m\lambda$$

where *m* is the diffraction order. When the light is blazing at +1 diffraction order, a relationship between the incident, diffraction, and blaze angle *γ* can be extracted as Equation (2):

(2) α−γ=β+γ

Then substituting Equation (2) into Equation (1), the relationship between wavelength, grating constant, incident angle, and blaze angle is expressed by Equation (3).

(3) $$\gamma =\frac{1}{2}\left(\alpha -\arcsin\left(\sin\alpha -\frac{\lambda }{d}\right)\right)$$

It is seen that the wavelength of the grating is closely related to blaze angle and grating constant. Moreover, a spectrum will be generated at +1 diffraction order if a polychromatic light incident on the blazed grating in the same way, and any wavelength *λ* in the spectrum must satisfy the grating equation. Substituting sin *β* ≥ −1 into Equation (2), the reasonable range of grating constant is obtained by Equation (4),

(4) $$d\geq \frac{\lambda_{\max}}{1+\sin\alpha }$$

where *λ*_max_ denotes the maximum wavelength in the spectrum.

As the blazed grating is applied in the miniature near-infrared spectrometer whose spectrum range is from 800 nm to 2500 nm. The incident angle α is fixed at 14.1° as the requirement of optical path in the spectrometer. According to Equation (4), grating constant must satisfy *d* ≥ 2 μm. However, a large grating constant will result in a low spectral resolution. Therefore, considering the spectral range, spectral resolution, and lithography resolution, the grating constant is determined to be 4 μm. The blaze wavelength is derived from the thumb rule that the diffraction efficiency drops to 50% of its peak value at 2/3 *λ_b_* and at 9/5 *λ_b_*, where *λ_b_* is the blaze wavelength [30]. The wide spectrum ranges from 800 nm to 2500 nm should contain the range from 2/3 *λ_b_* to 9/5 *λ_b_* to get high diffraction efficiency. So, the blaze wavelength is calculated as 1200–1389 nm. Substituting it into Equation (3), the desired blaze angle is 8.7°–10.0°.

### 2.2. Optimization of the Blaze Angle

**Figure 2:**
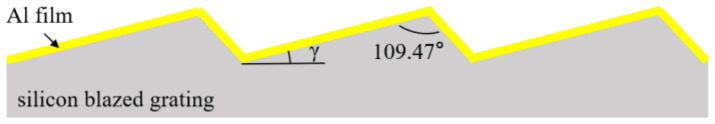
The grating model for diffraction efficiency simulation.

PCGrate is the software used for the exact calculation of diffraction efficiency by the accurate boundary integral equation method. In this work, the diffraction efficiency curves under different blaze angles are simulated using PCGrate Demo v.6.4 to get optimal blaze angle. Figure 2 shows the grating model for simulation. The gray area is the planar silicon-blazed grating with grating constant of 4 μm. To increase reflectivity, a 100 nm-thick aluminum (Al) film is added to cover the grating surface. Since the grating is fabricated by anisotropic wet etching process, the grating groove is formed by intersecting (111) faces and the apex angle of the grating is 109.47°. So, the border profile of the grating is defined by the blaze angle.

**Figure 3:**
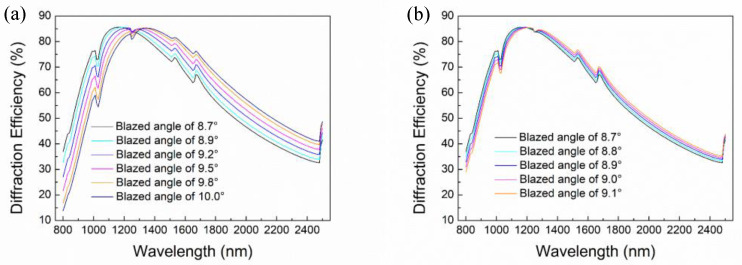
Diffraction efficiency of blazed gratings with blaze angle of (**a**) 8.7°, 8.9°, 9.2°, 9.5°, 9.8°, and 10.0°; (**b**) 8.7°, 8.8°, 8.9°, 9.0°, and 9.1°.

In order to preliminarily determine the range of optimal blaze angel, diffraction efficiency curves under blaze angle of 8.7°, 8.9°, 9.2°, 9.5°, 9.8°, and 10.0° are analyzed, as shown in Figure 3a. It can be seen that the diffraction efficiency increases rapidly and then decreases slowly as the wavelength increases. It gradually goes down in short wavelength and changes in the opposite direction in long wavelength as the increase of the blaze angle. Moreover, some anomalous jumps are clearly observed on these curves, which can be attributed to the exceeding 90° of the diffraction angle at a high order. The diffraction efficiency jumps into the lower orders when higher orders are diffracted into the grating surface and become evanescent. Through comparative analysis of diffraction efficiency at different blazed angles and different wavelengths, it is found that the blazed grating has higher diffraction efficiency when the blaze angle is 8.9°. The diffraction efficiency is higher than 32.84%. It means that the optimal blaze angle is around 8.9°. Then, the blaze angle is further optimized by setting blaze angle as 8.7°, 8.8°, 8.9°, 9.0°, 9.1° respectively. The diffraction efficiency curves are shown in Figure 3b. It shows that the diffraction efficiency is higher than 33.24% when the blaze angle is 8.8°, so the final optimized blaze angle is 8.8°. At this time, the lowest diffraction efficiency is 33.24% at wavelength 800 nm, while the maximum value is 85.62% at blaze wavelength 1180 nm.

**Figure 4:**
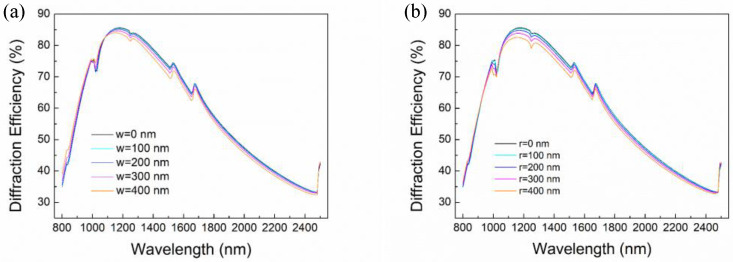
Diffraction efficiency with different (**a**) width of platforms; (**b**) radius of fillets.

However, a platform or fillet usually remains on the top of the grating groove during the blazed grating fabrication process, which induces stray light and degrades the diffraction efficiency. Therefore, it should be considered in the design process. For a clear view, diffraction efficiency under blaze angle of 8.8° varying with different platform and fillet size are also investigated, as shown in Figure 4. It can be seen that the diffraction efficiency gradually decreases as the platform becomes wider. Assuming the top of the grating is a platform, the diffraction efficiency at blaze wavelength of 1150 nm is 84.03% when the platform width is 400 nm, while it is 85.45% when the platform width is 100 nm. While for fillet, the diffraction efficiency at blaze wavelength is 82.54% and 85.39% respectively, when the radius is 400 nm and 100 nm. The result denotes that the diffraction efficiency indeed decreases with the increase of platform or fillet size, so it needs to be minimized in subsequent fabrication process. Furthermore, the effect of platform or fillet on diffraction efficiency becomes small when the width of the platform or fillet is less than 100 nm. Therefore, the platform width or the fillet radius of the blazed gratings should be sharpened to less than 100 nm to obtain high performance.

## 3. Fabrication and Characterization of Blazed Gratings

After the determination of the grating structure, it is important to control the groove shape during the next fabrication process. In this section, an improved process is proposed to fabricate the blazed gratings.

### 3.1. Experimental

**Figure 5:**
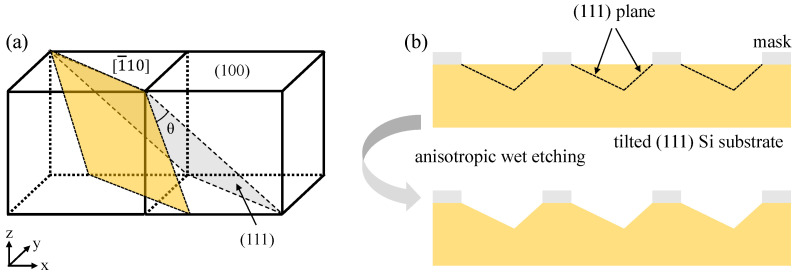
(**a**) The schematic diagram of tilted (111) silicon cutting; (**b**) formation of blazed grating groove by anisotropic wet etching.

To fabricate the sawtooth grating groove, the tilted (111) silicon wafer was used as the substrate. Figure 5a illustrates the schematic diagram of titled silicon wafer cutting. It was cut by (111) silicon ingot, which rotated a certain angle *θ* from (111) crystal plane to (110) plane along [$\bar{1}$10] orientation. The cutting angle *θ* is equal to the designed blaze angle *γ.* According to the characteristics of anisotropic wet etching, the grating groove was formed by the intersection of slowly etched surface (111) faces, as shown in Figure 5b.

**Figure 6:**
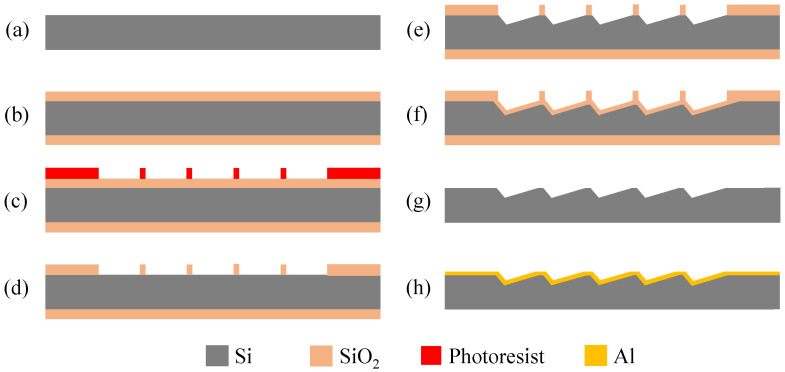
The fabrication flow of the blazed grating. (**a**) substrate preparation; (**b**) thermal oxidation of the substrate; (**c**) photoresist mask preparation; (**d**) SiO_2_ mask preparation; (**e**) anisotropic wet etching; (**f**) thermal oxidation of the grating; (**g**) SiO_2_ removing; (**h**) aluminum film deposition.

The microfabrication process of the blazed grating is depicted in Figure 6. First, the double sides polished, tilted (111) silicon wafer with cutting angle of 8.8° was used as the substrate (Figure 6a), whose thickness was 500 ± 20 μm, resistivity was 70–80 Ω·cm, and error of the cutting angle is ±0.5°. Then, a 300-nm-thick SiO_2_ layer was thermally oxidated on the wafer surface (Figure 6b). Next, a photoresist layer was coated on the SiO_2_ layer and a series of 500-nm-wide photoresist stripes which had 4 μm period and paralleled to the [$\bar{1}$10] direction was fabricated by stepper lithography (Figure 6c). The SiO_2_ layer was etched by reactive ion etching (RIE) technique and buffered oxidate etch (BOE) to form etching mask of gratings (Figure 6d). Subsequently, the grating groove was shaped by anisotropic wet etching (Figure 6e). An additional oxidation of gratings without removing the SiO_2_ etching mask was purposely taken to further decrease the platform on grating top (Figure 6f). The desired grating groove was obtained by removing SiO_2_ on both sides of the wafer (Figure 6g). Finally, a 100 nm-thick aluminum was deposited on the grating surface using magnetron sputtering to increase the reflectivity for spectral range of 800–2500 nm (Figure 6h).

### 3.2. Fabrication of Grating Etching Mask

**Figure 7:**
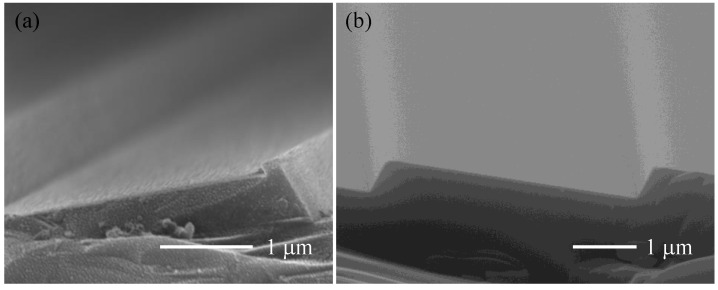
Grating grooves prepared under different grating mask preparation process. (**a**) only using RIE technique; (**b**) combining RIE technique and BOE wet etching technique.

SiO_2_ mask was used as etching mask of gratings in the experiment. In order to reduce the influence of platform on the performance of gratings, the SiO_2_ mask should be minimized. RIE and BOE isotropic etching was used to fabricate SiO_2_ mask and reduce its width. Generally, it was difficult to accurately etch 300 nm-thick SiO_2_ layer and stop on the Si surface only using RIE technique. If the SiO_2_ layer was overetched, the underlying Si substrate would be also slightly etched, forming a large nub on the top of the grating groove after anisotropic wet etching process, as shown in Figure 7a. The nub was also shaped by (111) plane and hard to be eliminated, so the diffraction efficiency is reduced because of introducing stray light and narrowing the blazed facet. Whereas, long-time wet etching in BOE could damage and cause peeling of the photoresist. Therefore, after RIE etching of SiO_2_ for 250 nm, BOE was applied to etch the remaining SiO_2_, and the etching would spontaneously stop at the interface of SiO_2_ and Si. The grating groove fabricated in this way is shown in Figure 7b, and the nub is successfully avoided.

**Figure 8:**
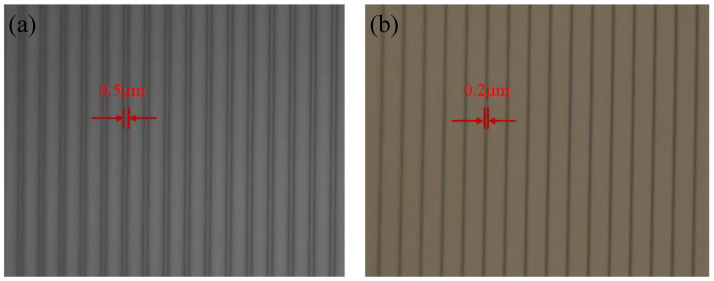
Images of (**a**) photoresist stripes before the etching process; (**b**) SiO_2_ stripes after photoresist removing.

Optimized parameters of RIE and BOE wet etching were investigated for good etching uniformity and small size etching mask. Sulfur hexafluoride (SF_6_) was used as the etchant gas, and oxygen (O_2_) was used as protection gas in the RIE process. A high SF_6_ flow and high etching power resulted in a high etching rate, which was set to be 30 sccm and 150 W respectively. As the O_2_ flow increased, anisotropy and uniformity of RIE etching were both improved. However, a small width of SiO_2_ stripes was needed in the experiment, which can be realized by isotropic lateral etching at low O_2_ flow. Moreover, high O_2_ flow would easily remove the photoresist, so O_2_ flow was set to be 5 sccm. After 180 s of etching by RIE, the thickness of the remaining SiO_2_ was 50nm, then it was overetched by BOE solution for 90 s. Images of photoresist stripes before etching process and SiO_2_ stripes after photoresist removing are shown in Figure 8. It could be seen that the width of the grating mask was narrowed from 500 nm to 200 nm.

### 3.3. Anisotropic Wet Etching of Blazed Gratings

**Figure 9:**
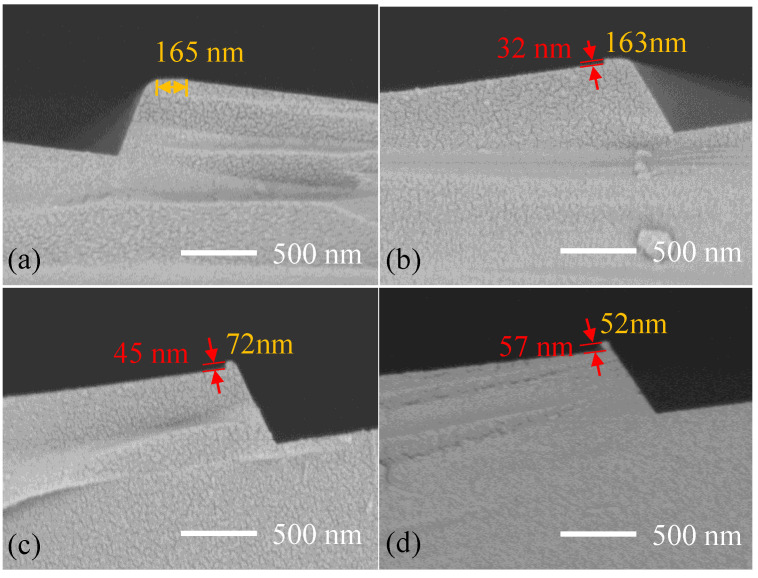
Grating grooves etched by different time. (**a**) 75 s; (**b**) 3 min; (**c**) 5 min; and (**d**) 7 min.

The gratings were prepared by anisotropic wet etching process. The exposed silicon was etched in the anisotropic etching solution at different rates according to the crystal direction. When the two (111) crystal planes intersected, the etching stopped, and the triangle blazed gratings were obtained. The blaze angle of the gratings was the tangent angle of tilted silicon wafer. For smooth grating surface, tetramethylammonium hydroxide (TMAH) solution with mass fraction of 25% was used in the experiment. The grating groove shapes at different etching time of 75 s, 3 min, 5 min, and 7 min were observed by scanning electron microscopy (SEM) after SiO_2_ mask removing, and the morphology of the platform on the top of the grating was analyzed. It was seen that a 165-nm-width platform was formed on the top of the grating due to the protection of a narrow SiO_2_ mask as the etching time was 75 s, as shown in Figure 9a. The rounded edge was caused by lateral corner cut corrosion. Then, a small nub emerges as the etching time increases, due to the over etching of the (111) planes and shaped by new (111) sidewalls. The height of the nub was 32 nm and the width was 163 nm after 3 min etching, as shown in Figure 9b. The nub became larger in depth and smaller in width with the increasing of etching time because of the constant etching of (111) planes, as shown in Figure 9c,d. When the etching time was 7 min, the width of the nub was 52 nm, and the height was 57 nm. However, the nub was difficult to eliminate further because the SiO_2_ stripes fell off as continuous etching. Moreover, the long-term wet etching led to an increase in surface roughness of the grating surface, which ultimately affected the diffraction efficiency. Hence, the suitable etching time was 75 s.

### 3.4. Oxidation Sharpening

**Figure 10:**
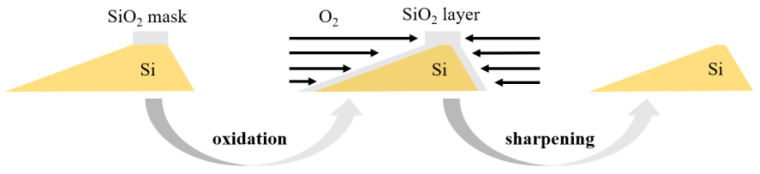
Schematic diagram of oxidation sharpening process.

After anisotropic wet etching process, an oxidation sharpening process was subsequently employed to further decrease the platform without removing the SiO_2_ etching mask. Figure 10 schematically displays the oxidation sharpening process. The grating samples were thermally oxidated for hours at 1050 °C, oxidation rate at the top of the grating groove was much lower than that of the groove sides since the SiO_2_ mask was served as a barrier layer. As a result, the thermal oxide layers are mainly grown at the sides of the groove. Subsequently, the oxidation layer was etched in BOE solution until the Si surface was hydrophobic, and the grating groove was effectively sharpened.

**Figure 11:**
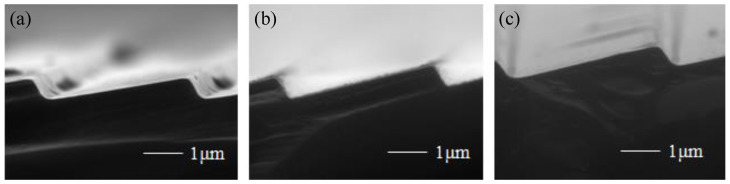
Grating grooves prepared under different oxidation time of (**a**) 1 h; (**b**) 2 h; and (**c**) 4 h.

The grating groove depended on the oxidation time. The grating samples was oxidated at 1050 °C for 1 h, 2 h, and 4 h, respectively, and the SEM images of the gratings after the oxide layer removing are shown in Figure 11. It can be seen that the platforms decreased and finally converted to a fillet. The radius of the fillet first decreased and then increased as the oxidation time increased. There was a 150-nm-wide platform on the grating top when the oxidation time was 1 h. When the oxidation time increased to 2 h, the platform turned to a fillet, whose radius was 50 nm. However, continued oxidation for 4 h caused the fillet to become larger, and the radius was about 130 nm. Therefore, the optimized oxidation sharpening time was 2 h.

**Figure 12:**
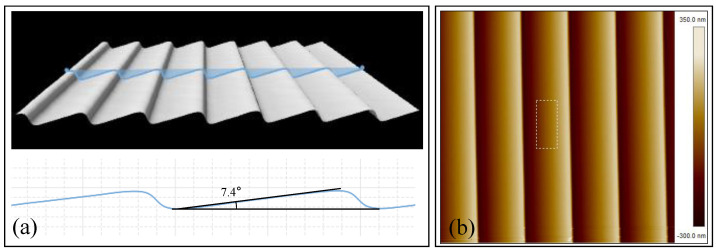
(**a**) Image of blazed grating profile; (**b**) AFM image of the blazed grating.

The grating profile measured by the optical profiler is shown in Figure 12a. The actual blaze angle was 7.4°, which had an offset of 1.4° to the designed value. This deviation mainly came from two sources. One was the cutting angle error of the tilted (111) substrate, another was the etching time difference between upper and lower surfaces. The atomic force microscopy (AFM) image of the blazed grating is also presented in Figure 12b. It could be seen that the measured root mean square (RMS) roughness of the grating surface with aluminum reflective coating was 0.477 nm, that proved the grating surface possessed great smoothness.

## 4. Application in Scanning Grating Micromirror

**Figure 13:**
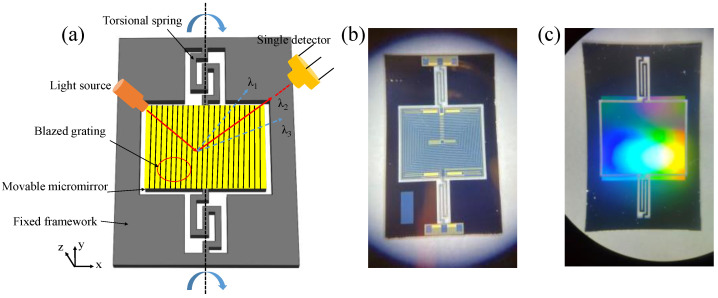
(**a**) Schematic diagram of the scanning grating micromirror; (**b**) frontside of fabricated scanning grating micromirror chip with actuated coils; (**c**) backside of fabricated scanning grating micromirror chip integrated with blazed gratings.

The fabricated blazed grating was integrated on the movable micromirror to form a scanning grating micromirror applying in the miniature near-infrared spectrometer. It could realize optical splitting and scanning at the same time. The schematic diagram of the scanning grating micromirror is shown in Figure 13. A compound light dispersed into lights arranging in order of wavelength to form a spectrum by the dispersion role of the integrated blazed grating. With the micromirror rotating around torsional springs, the incident angle continuously changed as well as the diffraction angle, resulting in scanning of the diffracted lights. Consequently, a single detector at a fixed position could be able to detect the diffraction spectrum. Applying this device in the near-infrared spectrometer can avoid using expensive near-infrared detection array and reduce the spectrometer size. Using standard MEMS fabrication technology, the blazed grating was integrated on the backside of the scanning micromirror to form scanning grating micromirror. The fabricated device is shown in Figure 13b,c.

**Figure 14:**
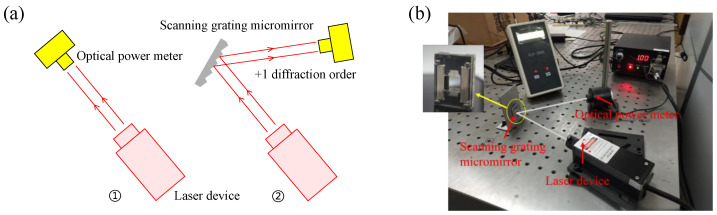
(**a**) Schematic diagram and (**b**) photography of the grating diffraction efficiency measurement setup.

The absolute diffraction efficiency is an important performance parameter for the scanning grating micromirror, which indicates the optical energy transfer capability. It is a measure of how much optical power is diffracted into a designated direction compared to the power incident onto the diffractive element, which is defined as:

(5) $$\eta_{\lambda ,m}=\frac{I_{\lambda ,m}}{I_{\lambda }}$$

where *I_λ,m_* is diffraction light power at diffraction order of *m*, and *I_λ_* is the incident light power. Figure 14a schematically showed the measurement for absolute diffraction efficiency. The set up mainly consisted of a laser device, an optical power meter, and a scanning grating micromirror. First, the laser was normally incident into the optical power meter to directly detect the incident laser power *I_λ_*. Then, the laser was incident into the center of the scanning grating micromirror with the incident angle of 14.1°, and the optical power meter was moved to detect the light power at +1 diffraction order *I_λ_*_+1_. According to Equation (5), the absolute diffraction efficiency could be calculated. Figure 14b shows the photographic image of the measurement set up.

Since the laser emitted TM-polarized wave in our test system, the measured results indicated as diffraction efficiency under TM polarization. Here, theoretical diffraction efficiency of blazed grating with blaze angle of 7.4° under TM polarization was obtained by PCGrate, and the theoretical values at given wavelength spots are given in Table 1. The actual diffraction efficiency was tested and compared to its corresponding theoretical value. The results showed high diffraction efficiency of the blazed grating but an error of 4–5% to the ideal value. This error was derived from the oxidation of Al reflective layer and dust adhesion on the grating surface in the test environment, which was acceptable.

**Figure 15:**
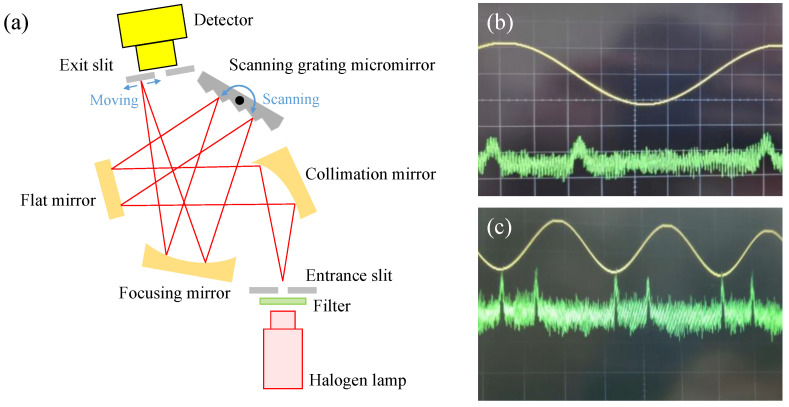
(**a**) Schematic diagram of diffracted wavelength range testing; (**b**) response of the photodetector to monochromatic light (**b**) 810 nm; (**c**) 2580 nm.

The diffracted wavelength range of the proposed scanning grating micromirror was tested with our previous NIR spectrometer setup [31]. As Figure 15a demonstrates, the light was emitted through a filter and an entrance slit. Then it was collimated by an off-axis parabolic mirror and reflected toward the scanning grating micromirror by using a flat mirror. The diffracted lights were reflected and focused on the detector’s plane through an exit slit. As the scanning grating micromirror tilted, the diffraction lights with varying wavelengths were scanned through the detector which gave the spectrum information for the incoming lights.

During the diffracted wavelength range measurement, a set of narrow band filters and a halogen tungsten lamp were used. The detector’s response over scanning process was amplified and recorded with an oscilloscope. The measured data with 810 nm (bandwidth of 10 nm) and 2580 nm (bandwidth of 50 nm) filters are given in Figure 15b and c respectively. The yellow line is the driving voltage for the rotation of scanning grating micromirror, and the green line is the spectral signal received by the detector. Two peaks of detected signal per period of driving voltage are observed since the diffracted light scans through the detector back and forth in a rotation period of scanning grating micromirror. The peaks on the recorded detector’s response (the green line) indicates that the proposed scanning grating micromirror has enough diffraction efficiency in the spectral range of 810–2500 nm for potential spectrometer application.

## 5. Conclusions

In this paper, a blazed grating applied in near-infrared scanning grating micromirror was designed, fabricated, and characterized. Based on the diffraction theory and diffraction efficiency simulation, the blaze angle and grating constant were determined as 8.8° and 4 μm, respectively. The effects of the platform and fillet on the diffraction efficiency were also investigated, showing a larger platform and fillet resulted in lower diffraction efficiency. However, the diffraction efficiency hardly decreased when the platform width or the fillet radius was less than 100 nm, which provided a target for controlling the defects on grating shapes in the fabrication process. By optimizing RIE and wet etching process, the SiO_2_ mask width was narrowed from 500 nm to 200 nm. The blazed grating was fabricated by anisotropic wet etching combined with high temperature oxidation sharpening process using tilted (111) silicon substrate. The platform was minished to a fillet with radius of 50 nm by optimizing etching time and oxidation sharpening time. The grating grooves were analyzed by optical profiler and AFM, showing the fabricated blaze angle was 7.4° and the RMS roughness of the grating surface with aluminum reflective coating was 0.477 nm. Finally, the blazed grating was integrated in scanning micromirror to form scanning grating micromirror using MEMS fabrication technology, which can realize both optical splitting and scanning. The testing results show that the scanning grating micromirror has high diffraction efficiency in the spectral range of 810–2500 nm. The proposed design and fabrication method of blazed gratings are promising to obtain high-performance blazed gratings applied in spectral analysis instruments.

## Figures and Tables

**Table 1:** The measured results of the diffraction efficiency at +1 diffraction order.

λ (nm)	η_TM (experimental)_	η_TM (theoretical)_	error
808	56.79%	61.52%	4.73%
1064	86.35%	90.87%	4.52%
1550	67.73%	72.22%	4.49%

## Data Availability

Data available on request due to restrictions, e.g., privacy or ethical. The data and material presented in this study are available on request from the corresponding author.
